# The Perfect Storm of Information: Combining Traditional and Non-Traditional Data Sources for Public Health Situational Awareness During Hurricane Response

**DOI:** 10.1371/currents.dis.d2800aa4e536b9d6849e966e91488003

**Published:** 2013-12-16

**Authors:** Kelly J. Bennett, Jennifer M. Olsen, Sara Harris, Sumiko Mekaru, Alicia A. Livinski, John S. Brownstein

**Affiliations:** Office of Emergency Management, Division of Fusion, U.S. Department of Health and Human Services, Washington, DC, USA; Office of Emergency Management, U.S. Department of Health and Human Services, Washington, DC, USA; Office of Emergency Management, Division of Fusion, U.S. Department of Health and Human Services, Washington, DC, USA; Boston Children's Hospital, Emergency Medicine, Boston, MA, USA; Office of Administration, National Institutes of Health, Division of Library Services, Bethesda, MD, USA; Boston Children’s Hospital, Harvard Medical School, Boston, MA, USA

## Abstract

<b>Background:</b> Hurricane Isaac made landfall in southeastern Louisiana in late August 2012, resulting in extensive storm surge and inland flooding. As the lead federal agency responsible for medical and public health response and recovery coordination, the Department of Health and Human Services (HHS) must have situational awareness to prepare for and address state and local requests for assistance following hurricanes. Both traditional and non-traditional data have been used to improve situational awareness in fields like disease surveillance and seismology. This study investigated whether non-traditional data (i.e., tweets and news reports) fill a void in traditional data reporting during hurricane response, as well as whether non-traditional data improve the timeliness for reporting identified HHS Essential Elements of Information (EEI). <b>Methods:</b> HHS EEIs provided the information collection guidance, and when the information indicated there was a potential public health threat, an event was identified and categorized within the larger scope of overall Hurricane Issac situational awareness. Tweets, news reports, press releases, and federal situation reports during Hurricane Isaac response were analyzed for information about EEIs. Data that pertained to the same EEI were linked together and given a unique event identification number to enable more detailed analysis of source content. Reports of sixteen unique events were examined for types of data sources reporting on the event and timeliness of the reports. <b>Results:</b> Of these sixteen unique events identified, six were reported by only a single data source, four were reported by two data sources, four were reported by three data sources, and two were reported by four or more data sources. For five of the events where news tweets were one of multiple sources of information about an event, the tweet occurred prior to the news report, press release, local government\emergency management tweet, and federal situation report. In all circumstances where citizens were reporting along with other sources, the citizen tweet was the earliest notification of the event. <b>Conclusion:</b> Critical information is being shared by citizens, news organizations, and local government representatives. To have situational awareness for providing timely, life-saving public health and medical response following a hurricane, this study shows that non-traditional data sources should augment traditional data sources and can fill some of the gaps in traditional reporting. During a hurricane response where early event detection can save lives and reduce morbidity, tweets can provide a source of information for early warning. In times of limited budgets, investing technical and personnel resources to efficiently and effectively gather, curate, and analyze non-traditional data for improved situational awareness can yield a high return on investment.

## Background

Isaac existed as a tropical cyclone through most of its trajectory until nearing southeastern Louisiana. A few hours before landfall on August 28, 2012, Isaac became a category one hurricane.^1^ Southeastern Louisiana and southern Mississippi experienced severe inland flooding and extensive storm surge, particularly in Plaquemines and St. Bernard Parishes in Louisiana.^1^

Public health concerns associated with hurricanes vary during and after a storm. As a storm comes ashore the majority of deaths and injuries are attributed to drowning and blunt trauma as a result of structural collapse or attempts to secure potential projectiles in high wind conditions.^2^ Post-landfall injuries and deaths are generally linked to gastrointestinal illness, falling trees, improper use of alternative power sources, and trauma related to clearing debris.^2^ Additionally, there may be an increase in illness due to exposure to storm debris, a lack of hygienic living conditions, and contaminated water supply due to flood waters.^3^^,^ 4 ^,^ 5 Long term power outages or interruptions result in several health hazards including concerns with food safety and carbon monoxide poisoning.^6^^,^ 7 Finally, the emotional impact from disasters such as a hurricane can be long-lasting and wide-reaching.

Prior to Hurricane Isaac making landfall in the continental United States, federal emergency support functions were activated, including the public health and medical support functions led by the Department of Health and Human Services (HHS). In coordination with state and local emergency managers, the role of HHS as the lead federal agency for medical and public health response and recovery includes: providing timely, life-saving public health and medical infrastructure; behavioral health care for survivors and responders; care for the medical special needs population; mortuary assistance; and veterinary care.^8^ A key component to responding to and providing life-saving federal resources to a disaster-impacted area in a timely manner is having good situational awareness about the unfolding event including its immediate aftermath.

Situational awareness is defined within the National Response Framework as “the ability to identify, process, and comprehend the critical elements of information about an incident”.^9^ A cornerstone to responding is having situational awareness with regards to what is happening ‘on the ground’. It requires understanding the current situation and potential hazards and forecasting the ensuing risks and repercussions for the responders and affected community.^10^ Relevant and timely information are necessary to inform situational awareness. Good situational awareness is necessary for effective and rapid decision-making to direct response and recovery activities.^11^

Both traditional and non-traditional data can inform situational awareness. For example, during a disaster response to a hurricane, traditional data sources can include situation reports and press releases. Federal situation reports can provide a daily or twice daily snapshot of the engaged federal resources and assets, status of the response, and on-ground needs to be addressed. Non-traditional data sources include news reports and social media (e.g., Twitter, YouTube, Instagram). Twitter has become a popular platform for citizens, news agencies, government officials, and others to provide and receive information during time-sensitive situations like hurricanes.^12^

Non-traditional data has been used in other fields (e.g., disease surveillance and seismology) for improved situational awareness. Several works have shown a time correlation between trends in non-traditional data and official case data, with the non-traditional data available days to sometimes months earlier than official reports.^13^^,^ 14 ^,^ 15 ^,^ 16 News reports have been used for real-time disease outbreak tracking. The HealthMap platform collects, curates and processes news reports in fifteen languages and from numerous international sources to provide a visual tool for real-time tracking.^17^ While developed to monitor infectious disease outbreaks, the underlying software algorithms and machine-learning techniques have proven flexible enough to expand to non-disease monitoring like natural disasters. HealthMap collects data through public news sources, expert-curated discussions, validated official reports, and crowd-sourced self-reports via their Outbreaks Near Me mobile app and web submissions. Further, Twitter has been used as a sensor to detect earthquakes and subsequent alerts sent out prior to official notifications, based on an algorithm that monitors tweets for earthquake-related information.^18^ While non-traditional data has been shown to provide timely information and warning in some fields, little research has examined the role of non-traditional data for improved situational awareness and timeliness of reporting during a hurricane response.

An opportunity exists for non-traditional data sources to augment the traditional data currently collected and analyzed by HHS during a hurricane response. A HHS pre-determined list of hurricane Essential Elements of Information (EEIs) guides what information is critical, who is responsible for the data collection, and the frequency of reporting.^8 ^These data are intended to provide decision-makers with better situational awareness. Table 1 lists some of the HHS EEIs.

**Table 1: Selected HHS EEI Categories:** 

HHS EEI Categories
Status of critical infrastructure (i.e., hospitals, nursing homes, mental health clinics)
Status of shelters
Property damage in affected area and casualties (including fatalities)
Status of medical special needs populations
Injury/disease surveillance and outbreaks
Mandatory evacuations and relocation assistance
Medical assistance required with Urban Search and Rescue Teams
Blood product support
Vector-borne disease threat
Potable water status
Environmental conditions (including contamination)
Mortuary affairs/victim identification support (upon state and local government request)

In this retrospective study, following the HHS response to Hurricane Isaac, traditional data sources (i.e., federal government situation reports and state press releases) and non-traditional data sources (i.e., citizen and local government/emergency management (EM) tweets and news reports) were evaluated based on their relevance to the pre-determined EEIs to determine whether non-traditional data improve situational awareness by filling a void in traditional data reporting and whether non-traditional data improve the speed for which some EEIs were reported. EEIs are designed to provide and/or improve situational awareness regarding a given incident, as well as to understand the status of public health functional areas.^8^

## Methods

During the Hurricane Isaac response, the HHS Office of the Assistant Secretary for Preparedness and Response (ASPR) conducted near-real time review and analysis of data from Twitter and the news for information that could help address some of the EEIs. Analysts used Boolean searches, hashtags (e.g., #isaac), and targeted Twitter lists consisting of local news and local government/emergency management accounts to filter out large amounts of social media noise and focus on predetermined EEI categories. The terminology used for the Boolean searches was regularly updated throughout the course of the Isaac response in reaction to changing situations on the ground and informational needs. The most basic searches began with storm terminology linked with EEI terminology like “hospital”, “medical center”, “nursing home”, “carbon monoxide”, etc. An example of one of the initial search strings was *(Isaac OR hurricane OR “tropical storm”) AND hospital*. Both the Twitter lists and the Boolean searches were loaded into a social media dashboard. Twitter lists and Boolean searches were easily displayed side-by-side in separate panels on a single screen where analysts viewed tweets in real-time. Further, through a project with HealthMap, analysts reviewed pre-established hurricane-specific news report feeds set up to refresh hourly using vocabulary related to the EEI categories provided by HHS/ASPR.

Because initial data collection occurred in near-real time researchers cannot determine the exact number of news articles and tweets that were reviewed. Retrospective analysis using the Topsy Pro Public Sector Analytics^TM ^(Topsy) tool found that nearly one million tweets referencing the terms *Isaac, hurricane *or* tropical storm* were sent in the United States between August 27, 2012 and September 5, 2012. The number of tweets reviewed by ASPR analysts would likely fall below that number due to the additional search parameters used in the Boolean searches. Monitoring of news and social media during the course of the Hurricane Isaac response resulted in 143 tweets and news articles flagged as potentially relevant to the EEI categories. Following the HHS Isaac response, these 143 Twitter and news data collected by the analysts were consolidated into a table created in Microsoft Office Excel 2007 (Microsoft, Redmond, WA). This table contained the original data from which this study was developed.

***Dataset***

A data extraction table was created to consolidate the Louisiana-only findings from across the following data sources investigated for this study: Twitter, news, press releases, and situation reports (Table 2). The period of investigation for this study was August 27, 2012 through September 5, 2012. The data sources were analyzed for information that aligned with the HHS EEIs for hurricane response by research team members (KB, JO, SH, and SM).

**Table 2: Hurricane Isaac data sources:** 

Data Source	Description of Source	Type of Data Source
Situation report	Official report about an incident or event with verified information and explicit details from HHS/ASPR, the Department of Homeland Security National Operations Center, and the Department of Homeland Security/Federal Emergency Management Agency (FEMA).	Traditional
Press release	Official information provided to news organizations for dissemination. Press releases were from the City of New Orleans, the Governor’s Office of Homeland Security and Emergency Preparedness, the Office of the Governor of Louisiana, and the Louisiana Department of Health and Hospitals.	Traditional
News report	News reports captured in the HealthMap HHS Hurricane hurricane-specific news feeds. These feeds were developed in collaboration between HHS/ASPR and HealthMap in August 2011 to provide public health and medical news information during hurricanes.	Non-Traditional
Local government/EM tweet	Information reported on Twitter by local government officials providing updates, warnings, and public health messages. The local government/EM Twitter accounts included the Louisiana Governor, the Louisiana Governor’s Office of Homeland Security and Emergency Preparedness, the City of New Orleans, and Louisiana National Guard.	Non-Traditional
News tweet	Information reported on Twitter by news organizations or individual users who state affiliation with a news organization in their profiles. The news tweets are limited to those originally identified by HHS/ASPR during Hurricane Isaac response. The news tweets were primarily from local New Orleans and Baton Rouge news stations.	Non-Traditional
Citizen tweet	Real-time information publically posted on Twitter by private individuals who do not represent a news agency or the government. The citizen tweets are limited to those originally identified by HHS/ASPR during Hurricane Isaac response.	Non-Traditional

Each health-related data item was assigned a unique ID and the following information was recorded: data type (situation report, press release, tweet), data source, document name (if applicable), report date, report time, location, text of the data item, and URL (if applicable). Once the data extraction table was complete, each unique health-related data item was reviewed by members of the research team (KB, JO, SH), and a general EEI category was assigned. After reviewing the data table results, the following EEI categories were identified in this study: carbon monoxide poisoning, blood supply shortages, environmental hazards, medical special needs at-risk, fatality, and mandatory evacuations.

After the EEI categories were identified, the final dataset excluded: findings about locations outside of Louisiana; public messaging or sentiment; status of critical infrastructure; urban search and rescue; and potable water status.

***Identification of Unique Events***

For this study, an event was defined as an occurrence which may require targeted response to protect life or the public’s health within the larger scope of overall Hurricane Issac response. Next, researchers identified two EEI categories for which a total case count was imperative to track: carbon monoxide poisoning and hurricane-related fatalities. All data related to these two EEI categories were considered as part of a single “carbon monoxide poisoning” event or the “fatality” event.

EEI categories for which case counts were not relevant were also reviewed. For each data item, the event was compared and if the event occurred at the same location, these data were linked together and assigned a unique event identification number (Figure 1). If there were similar, but unrelated, EEI events that occurred in separate locations then each data item reporting a distinct location was identified as a unique event. For example, there were two reported instances of critical blood needs in Louisiana during Hurricane Isaac (one in Lake Charles and one in Terrebonne Parish). These two instances were each considered to be a unique event as the hazard occurred in two different locations.

**Flow diagram for identifying unique events:**
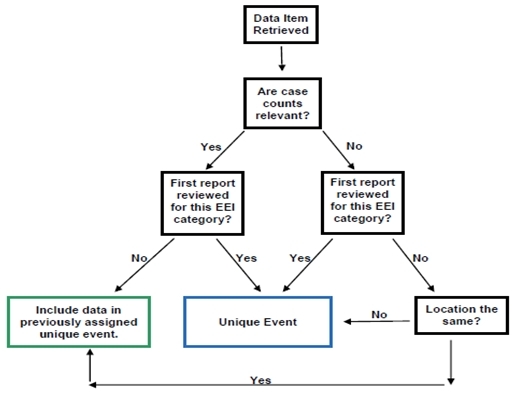

To understand which data sources provided information about each unique event, a unique event table was developed in Microsoft Excel to identify which sources reported on each event. An assessment was also done to determine which events had multiple reports as compared to only single source reporting.

***Timeliness Analysis of Unique Events***

An important question in this study was whether some data sources provided public health and medical-related information faster than other sources. To determine timeliness in reporting, the researchers analyzed reporting times for each unique event by source where more than one data source contributed findings. For each data item, the researchers rounded to the hour if there were 29 minutes or less, and up to the next hour if there were 30 minutes or more. If a single source reported multiple times on an event, the first time the source reported was used for these calculations.

## Results

The final dataset identified sixteen unique events. Mandatory evacuations accounted for the highest number of unique events (4), followed by medical special needs events (3). Of these sixteen events, six were reported by only a single data source, four were reported by two data sources, four were reported by three data sources, and two were reported by four or more data sources (Table 3). Data from traditional data sources was reported in six of the unique events (38%), while data from non-traditional sources was reported in fourteen of the unique events (88%). For all of the unique events, the data source(s) varied, even across the same EEI categories.

All of the mandatory evacuation events were reported in at least two sources, while all of the medical special needs events were only reported by a single source. One event (ID-13) had four data sources of content: news report, news tweet, local government/EM tweet, and press release, while one event (ID-12) was reported in five of the six source types that were included. Of events that only had one source, the sources were distributed across all data types except situation reports. If an event was identified in a situation report, it was also reported in at least one other data source. For events with two sources identified, there was no consistent combination of which two data sources were reported.

**Table 3: Hurricane Isaac unique events**

**Figure:**
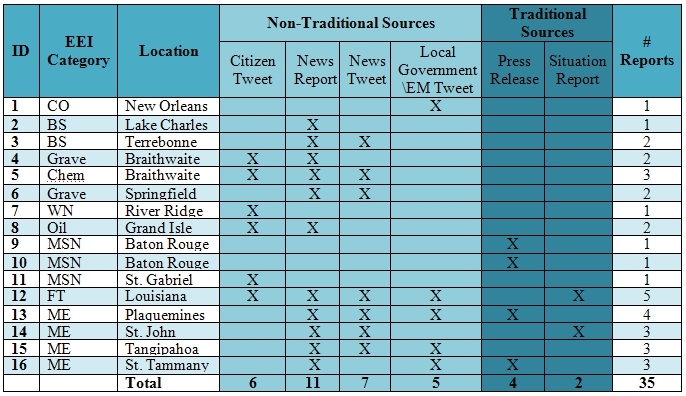
**Legend:** CO=carbon monoxide poisoning; BS=blood supply shortage; Grave=grave disinterment; Chem=chemical spill; WN=West Nile Virus; Oil=oil spill; MSN=medical special needs; FT=fatality; ME=mandatory evacuation

Second, the researchers looked for commonality across the events and data sources. Among the unique events that were analyzed, information came from diverse data sources: six events from citizens (via Twitter), eleven events from news organizations (via Twitter or news websites), seven events from local governments (via Twitter and press releases), and two via federal situation reports. Citizens were a source of information in most (four out of five) environmental events, while press releases were the only source for two (of three) medical special needs events. Of the four mandatory evacuation events included, news tweets and reports provided information on all four of the evacuations while local government/EM tweets provided details about three events.

For the third phase of analysis, the timing across different data sources was assessed. While there are many events where the same data source reported the event and then provided updates, the timeliness assessment focused on only events for which more than one type of data source referenced the event (n=9). Figure 2 shows nine of the ten unique events where two or more data sources reported about the event (with ID-12 excluded and analyzed in further detail below). In all nine events examined, the first report about the event was captured in a tweet. For five of the events where news tweets were one of multiple sources of information about an event (ID-3, 6, 13, 14, and 15), the tweet occurred prior to the news report, press release, local government/EM tweet, and situation report. In events where citizens were reporting along with other sources (ID-4, 5, and 8), the citizen tweet was the earliest notification of the event. For ID-14, the situation report content was the last source of data, by almost two days after the news tweet. There was also a lengthy time difference of over seven days between the citizen tweet and the news report about grave disinterment (ID-4).

**Timeliness analysis by event and source for unique events:**
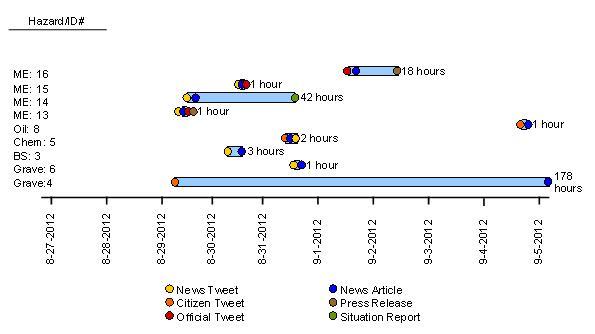
**Legend:** ME=mandatory evacuation; Oil=oil spill; Chem=chemical spill; BS=blood supply shortage; Grave=grave disinterment

For two event types, carbon monoxide poisonings and fatalities, HHS/ASPR captured case counts. For carbon monoxide poisoning (ID-1), only one data source (non-traditional) provided informational updates (a local government\EM Twitter account). This source provided an initial report of cases of carbon monoxide poisoning on August 31, 2012, and proceeded to update the case count number two more times over the next 28 hours. For fatality tracking, a prevalence curve is displayed in Figure 3 demonstrating the relationship between reported fatalities (ID-12) and data sources over time. The news reports (non-traditional) provided the fatality count in advance of any other sources, while situation reports (traditional) did not begin reporting any fatalities until September 3, 2012 with a first report of five fatalities (Figure 3). Press releases did not provide any fatality counts throughout the Hurricane Isaac response.

**Fatality reporting news reports vs. situation reports:**
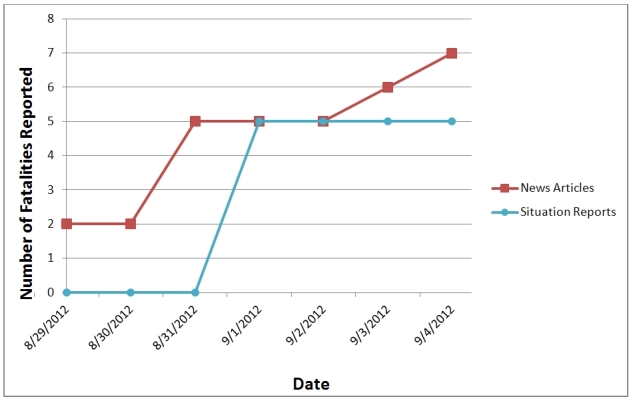

## Discussion

In this paper, researchers provided an examination of traditional and non-traditional data sources for event reporting during the HHS/ASPR Hurricane Isaac response. With such a broad range of data collection categories in the HHS EEI, the results indicate that multiple sources of non-traditional and traditional data are required for decision-makers to have an improved understanding of what is happening ‘on the ground’ during a hurricane. In several instances, non-traditional data sources provided the only information about certain events including all reports about blood supply issues, disinterred graves, a possible chemical leak, and an oil hazard. Of these nine events in which non-traditional data sources were the first reports, citizen tweets provided the first indication of an event for three (disinterred graves, possible chemical leak, and an oil hazard).

Situation reports serve the purpose of official reporting about an area, incident, or event with verified information and explicit details; however, their intended purpose is not to provide near-real time data. The situation reports can lack timeliness as officials confirm data that is collected from the hurricane-impacted area due to the need for including only verified data. Only two unique events in this study had data from situation reports (ID-12 and 14) and the reporting lag was up to 42 hours. The timeliness in reporting across the traditional and non-traditional data showed that non-traditional data (i.e., news reports and Twitter) is quicker to be reported. These results align with other research where reporting times were examined for traditional and non-traditional data sources.^15^ Twitter has become a platform for citizens, news agencies, government officials, and other groups to share and receive information in near-real time. During a hurricane response where early event detection can save lives and reduce morbidity, Twitter could serve as an additional source of information for establishing situational awareness or for an early warning of potential problems ahead. To augment official reporting with near-real time data, HHS EEIs might benefit from identifying which EEI categories may have data available earlier from non-traditional data sources and which EEI categories require verified data found in traditional data sources.

For both carbon monoxide poisoning and fatalities, the methodological approach was different as the intent was to gather an updated count of instances over the response time period. The unique nature of these two EEI categories required an individual assessment of the various data source(s) reporting, as well as the count of instances (poisonings or fatalities) per source over date and time. In this study, news information (i.e., non-traditional data) provided in the HHS hurricane-specific news feed from HealthMap provided the more timely case count numbers over the course of the response. The prevalence curve for fatalities (Figure 3) clearly showed the news sources were timelier, and potentially more complete, reporting of fatalities. As the reporting of fatalities was delayed in traditional sources of data (i.e., situation reports), news reports may be a better source for establishing early situational awareness until official fatality reports are available.

The number of fatalities resulting from a hurricane is important to be aware of if federal assistance is requested to help with victim identification and mortuary affairs. Further, understanding the causes of death can assist with public health messaging around health hazards during and after a hurricane. Boak’s 2007 research indicated that fatality tracking is improved by using data that is freely available on the internet (i.e., death notices in online newspapers).^19^ Therefore, an opportunity exists for improved fatality tracking and situational awareness through monitoring non-traditional data sources such as news reports; however, further research is needed.

Carbon monoxide poisoning during hurricane response is typically attributed to inappropriate use of generators.^7^ Escalating cases of carbon monoxide poisoning may indicate that public health messaging about safe generator use needs to be increased and targeted to at-risk groups. It may be imperative to inform the federal medical teams deployed to a location about the increasing cases of carbon monoxide poisoning so they are prepared to look for signs and symptoms of carbon monoxide poisoning with incoming patients. In this study, the only source of information about carbon monoxide poisonings was found in non-traditional sources via NOLA Ready, the City of New Orleans’ Emergency Alert System. The NOLA Ready twitter account regularly updated a running count of carbon monoxide cases in the city with the final number of cases reported being twenty.

Identifying the information needs or gaps, developing a strategy for examining non-traditional data sources, and incorporating this data with traditional data can improve a response organization’s awareness of the situation ‘on the ground’ in a timelier manner. In times of limited budgets, investing technical and personnel resources to efficiently and effectively gather, curate, and analyze non-traditional data for improved situational awareness can yield a high return on investment. Through building tailored search queries and lists to target health hazards identified before a disaster response, this can help to focus a broad social or news media search into a quicker, targeted and more manageable activity. Another key step is to pre-determine EEIs for each type of disaster, as well as a list of key terms (including misspellings and abbreviations) so that analysts are prepared once a disaster occurs.

Further, as “all disasters are local”,^20^ it is important to try to quickly identify top voices in the region, whether that be news stations, websites, or social media personalities. When looking for potential health hazards in non-traditional data sources, it is important to have an understanding of the norm in the impacted community. The ability of an individual to identify abnormalities is best performed when the person has a familiarity with his/her surroundings. This phenomenon of understanding “normal” applies to self-tracking,^20^^,^ 21 and can be applied more broadly to an environment. In addition, local community members are likely to be the first to report the out-of-ordinary events or anomalies that are not included on any predetermined list of potential health hazards (e.g., HHS EEI). Therefore, gathering information provided by local news organizations, local government, and local community members on Twitter (or other social media sources) will provide a greater and more complete awareness of the disaster situation in their communities rather than relying solely on federal data sources. As seen with ID-4, 5, and 8 during Hurricane Isaac, this was particularly true with perceived environmental hazards where citizens reported abnormalities in their communities (grave disinterment, chemical leak, and oil spills) before any of the other data sources whether traditional or non-traditional.

Gaining and maintaining situational awareness at the federal level during a hurricane response can be challenging on many levels. While Twitter has the potential to provide insight into what is happening ‘on the ground’, the spread of misinformation, fears about rumors, protecting privacy, and Internet connectivity issues put monitoring non-traditional data sources as secondary or even tertiary options during an emergency disaster response. Concerns over the ability to verify information collected via Twitter are understandable and can be addressed through careful monitoring and the use of established rules of validity. Tweets sent from citizen accounts should be approached with greater caution and require a greater level of verification on the part of the analyst. However, following local government/EM on Twitter can provide valid information for reports as the information shared is from a trusted and verifiable source. Further, information overload may result from introducing non-traditional data collection and analysis into current data collection plans from traditional sources.^22^ This data deluge can make it a challenge for decision-makers to make good decisions if they are overwhelmed with the data. Organizations planning to monitor non-traditional data sources should utilize tools that allow for automated filtration, deduplication, or characterization. Platforms like HealthMap that use automation and machine learning organize the deluge of data allowing for more efficient review by team members. For example, the news reports were automatically assigned a geo-location as they entered the system. Additionally, these kinds of tools support additional tagging that can flag an individual report as relating to a particular kind of EEI once the system has been trained.^17^ Hurricane impacts are different depending on the geography and demography of communities affected. A larger hurricane could result in a larger impacted area, possibly spanning multiple states along the United States eastern seaboard and Gulf of Mexico. As the size of the impacted area grows, the monitoring of non-traditional data sources becomes much more complex and time-consuming as information flows in from a multitude of non-traditional and traditional data sources and localities. Additionally, the communication methods and use of Twitter and other social media platforms by local government officials and news organizations will vary by location thereby making monitoring more of a challenge due to the need to monitor more than one state. The level of public engagement via Twitter can vary based on a variety of social and economic demographics.^22^^,^ 23 Because of this, the amount of relevant information gained from local citizens via Twitter will not be uniform for each storm.

Non-traditional data sources are not the ultimate panacea and have limitations inherent to each source type. One key limitation of using news data is that what stories get published is the result of competition for space and sensational events which may get more coverage due to reader interest rather than newsworthiness.^24^^,^ 25 The "salience" of news is determined by unexpectedness, proximity, conflict, discrepancy, prominence, or celebrity status of involved people.^25^^,^ 26 In terms of Twitter, one key limitation is that perceived severity and intense news coverage are likely factors that have been shown to dictate tweet posting activity.^26^ Yet analysis of a blend of traditional and non-traditional data during the response to a hurricane can provide better situational awareness than traditional data alone. While HHS has its specific mission and role to fulfill when activated to respond to a hurricane, other organizations involved in hurricane response activities may look to non-traditional data sources to fill their information gaps using their own pre-defined categories.

## Limitations

There were several limitations with this study. Some of the data collection was done retrospectively (situation reports, press releases). Twitter and news data was collected in real-time during the course of the response. However, researchers recognize that real-time monitoring does not guarantee a full collection of every relevant tweet or report. Further, collecting information from Twitter and news media in real-time during an event can be challenging due to the quick decision-making required by analysts to identify potentially relevant information from these sources before the larger scope of the event is fully understood.^23^^,^ 27 To account for this, researchers also retroactively reviewed the full HealthMap feed and tweets from selected local government/EM accounts for the response time period. A full retrospective Twitter analysis during the Hurricane Isaac time period was not conducted due to resource requirements. Data that was captured as a citizen tweet may not have originated with the citizen, but rather, may have originated with a news organization or government agency. Further, the sample size of 16 events is small, and the number of cases for the carbon monoxide poisoning and fatality events were low. Due to the low number of reports that included content from traditional data sources, no assessment of sensitivity, specificity, or predictive value could be completed. These small sample sizes weaken the generalizability of the study findings. Finally, this study was not designed to identify reports by any source that would have been related to an EEI but were later found to be incorrect.

## Further research

This study looked at a single hurricane in a single location; and therefore the results may not be generalized across different disasters or locations. Future research on this subject could include a similarly structured study for other disasters, include additional non-traditional data sources (e.g., Instagram, Facebook, YouTube, etc.), complete a comprehensive retrospective analysis across all of the data sources, or use the news alert data already collected to enhance automated tagging and geo-locating capabilities to improve efficiency. Also, this study did not address how Twitter usage and behavior changes through the course of a disaster. Further research is needed to understand when Twitter users are most actively sharing potential public health concerns, whether it be during a disaster or its immediate aftermath. As this study did not find trends or patterns that indicated which data source is best for selected health categories, further research is needed to examine where these patterns may exist. Further research is needed to take lessons learned in this study and other research to create an all-hazards framework for using Twitter and news media for improved situational awareness in emergency response.

## Conclusion

Critical information is shared by citizens, news organizations, and official representatives daily and during a disaster or emergency. Consequently, non-traditional data should no longer be a secondary thought in disaster and emergency response. To have situational awareness for providing timely, life-saving public health and medical response following a hurricane, this study demonstrates that non-traditional data sources should augment traditional data sources to fill some gaps inherent in traditional reporting. Specifically, the researchers determined that nontraditional data (in the form of Twitter) provided key information in advance of all nine events for which multiple data sources reported. Examining non-traditional information sources during a hurricane response can identify potential public health and medical hazards prior to traditional reporting, thereby giving response agencies early warning to prepare for, respond to, and recover from health and medical-related hazards. In times of limited budgets, investing technical and personnel resources to efficiently and effectively gather, curate, and analyze non-traditional data for improved situational awareness can yield a high return on investment. Non-traditional data such as Twitter, news reports, and other digitally-contributed content should not replace traditional sources of emergency management information, but rather, it should provide additional sources of near real-time information for improved situational awareness and early event detection.

## Competing Interests

JSB holds equity stake in Epidemico, a company formed by Boston Children's Hospital to handle licensing of HealthMap data. The authors declare that no other competing interests exist.
